# Supplementary material for: The Effects of Deep Brain Stimulation on Balance in Parkinson’s Disease as Measured Using Posturography—A Narrative Review
Source: Brain Sci. 2025 May 21;15(5):535. doi: 10.3390/brainsci15050535 (PMC12109885; doi:10.3390/brainsci15050535)
Supplement: Supplementary file 1 [file brainsci-15-00535-s001.zip › brainsci-3636812-supplementary.pdf]

Table S1. Studies investigating the effects of STN-DBS alone on posturography

Author & Year	Static/Dynamic	Participants	Participant baseline details	Controls	Control details	Posturography conditions	Posturography metrics
Cani et al 2024	Static	33 PD	27% female<br>Median age 62.0 years<br>Median disease duration 11.0 years<br>36.5% tremor dominant, 45.5% akinetic rigid, 18% mixed<br>Mean LEDD 1,005 mg<br>Mean UPDRS III: OFF 41.5<br>Mean H&Y 2	n/a	n/a	*Quiet standing sway:*<br>4 trials of 30 secs; hard surface, EO & EC; using wearables<br><br>Med-OFF > 12 hours without levodopa, > 24 hours without long-acting medications<br>Med-ON 45-60 mins after levodopa<br>DBS-ON – 30 mins<br>DBS-OFF - 60 mins	Sway path<br>Ellipse area<br>Mean velocity (AP, ML)<br>95% power frequency acceleration (AP, ML)
Cherif et al 2024<br>*Directional*	Dynamic	10 PD + severe FoG	20% female<br>Mean age 59.5 years<br>Mean disease duration 11.6 years<br>Mean FoG-Questionnaire 25.7<br>Mean LEDD 1,396 mg<br>Mean UPDRS III: OFF 42.3, ON 9.9	10 HC	40% female<br>Mean age 55.3 years	*Gait initiation:*<br>20 trials of voluntary stepping from force platform<br>RCT of 1) central-STN; 2) posterior-STN; 3) ring-mode stimulation<br><br>DBS-OFF – 1 hours<br>Med-OFF > 12 hours	CoP displacement (AP, ML) of APAs before foot-off<br>APA duration<br>First step length, width & velocity<br>Double-stance duration<br><br>(& Principal Component Analysis of above factors)
Colnat-Coulbois et al 2005	Static	12 PD	42% female<br>Median age 58.5 years<br>Median disease duration 11.5 years<br>Median UPDRS III: OFF 48.5, ON 15	n/a	n/a	*Sensory Organisation Test (SOT):*<br>20s EO, 20s EC<br><br>Tested in DBS-/Med-ON only	Equilibrium Scores (ES) and Ratios (R)<br>Sway PL<br>Sway Area<br>Mean velocity (AP, ML)
De La Casa-Fages et al 2017	Static	16 PD	56% female<br>Mean age 59.6 years	13 HC	46% female<br>Mean age 60.9 years	*Quiet standing sway:*<br>3x 20s of 10 paradigms, including combinations of EO,	Sway PL<br>Sway Area

			Mean disease duration 15.6 years<br>Mean stimulation duration 32.1 months<br>Mean LEDD 859 mg<br>Median H&Y: OFF 3, ON 2			EC, wide/narrow base, cognitive task, holding weight, precision task, on one foot (R foot + L foot)<br><br>DBS-OFF >40 mins<br>Med-OFF >12 hours	Maximum CoP displacement (AP, ML)<br>Mean velocity<br>Peak velocity (AP, ML)<br>RMS of CoP displacement (AP, ML)
Fransson et al 2021	Dynamic	10 PD	10% female<br>Mean age 64.3 years<br>Mean disease duration 18 years<br>Median stimulation duration 37 months<br>Median LEDD 416 mg	25 "young"<br>17 "old"	*"Young":*<br>Mean age 25.1 years<br>*"Old":*<br>Mean age 71.2 years	*Balance perturbation:*<br>200 secs with randomized blocks of calf vibration, DBS ON/OFF with Med-OFF, EO/EC<br><br>DBS-OFF > 30 mins<br>Med-OFF > 11 hours	Body posture change
Guehl et al 2006	Static	7 PD	14% female<br>Mean age 57.3 years<br>Mean disease duration 12.9 years<br>Mean LEDD 1,053 mg<br>Mean UPDRS III: OFF 37, ON 11.4<br>Median H&Y: OFF 3.9, ON 2.8	n/a	n/a	*Quiet standing sway:*<br>1 trial 51.2s on static square atop a rolling cylinder; data with EO only<br><br>DBS-OFF > 30 mins<br>Med-OFF > 12 hours	Sway Area<br>Mean CoP displacement
Heß et al 2023	Dynamic	31 PD	29% female<br>Mean age 64.5 years<br>Mean disease duration 15.4 years<br>Mean stimulation duration 27.8 months<br>Mean UPDRS III: ON 14.6<br>Mean H&Y 2	30 no DBS PD;<br>30 HC	*No DBS PD*:<br>24% female<br>Mean age 68.2 years<br>Mean disease duration 6.55 years<br>*HC:*<br>63% female<br>Mean age 70.6 years	*Balance perturbation:*<br>4x trials each of left, right, backwards and forwards translations on force platform<br><br>Med-ON & DBS-ON only- times not given	*EMG:*<br>GM & TA muscle activity RMS<br>GM/TA co-contraction ratio<br>GM & TA time to muscle activation<br><br>*Force platform:*<br>CoP range

Krishnamurthi et al 2012	Dynamic	4 PD	25% female<br>Mean age 62.2 years<br>Mean disease duration 11.5 years<br>3 bilateral, 1 unilateral DBS<br>Mean LEDD 966 mg	n/a	n/a	*Target acquisition with leaning:*<br>data divided into *initiation, mid and hold phases* based on LoS. 4 stimulation conditions (optimal, moderate, low and OFF) with meds unaltered & 2 visual biofeedback conditions (a. COP; b. POP)<br><br>DBS change > 20 mins<br>Med-ON only	*Initiation & Mid phases:*<br>Peak/Mean Phase Velocities<br>Path Length<br>Time Taken<br><br>*Hold phase:*<br>Error<br>Unsteadiness<br>Entry count
Leodori et al 2023	Dynamic	6 PD	50% female<br>Mean age 66 years<br>Mean disease duration 17 years<br>Mean LEDD 416 mg<br>Mean UPDRS III: OFF 64, ON 49	n/a	n/a	*Balance perturbation:*<br>computerised posturography with Biodex system. Tested: a) DBS- worse side; b) DBS- best side; c) DBS- bilateral; d) DBS-OFF<br>Med-OFF/ON times not given<br>DBS-ON: testing at 20 mins after each setting started	Stability Index (SI)<br>Falls Risk Index (FRI)
Li et al 2020a	Dynamic	16 PD	50% female<br>Median age 60.3 years<br>Median disease duration 9 years<br>Mean LEDD 1,226 mg<br>Mean UPDRS III: OFF 59, ON 42	n/a	n/a	*Target acquisition with leaning:*<br>computer screen for biofeedback using TecnoBody PROKIN<br><br>DBS-ON only<br>Med-OFF > 12 hours (> 72 hours for DA)	Target acquisition (%)<br>Trunk swing angle standard deviation<br>Time to acquire target
Li et al 2020b	Dynamic	16 PD	50% female<br>Median age 60.3 years<br>Median disease duration 10.4 years<br>Mean LEDD 1,226 mg	20 PD + Meds	60% female<br>Median age 57.9 years<br>Mean disease	*Target acquisition with leaning:*<br>computer screen for biofeedback using TecnoBody PROKIN	Target acquisition (%)<br>Trunk swing angle standard deviation<br>Time to acquire target

			Mean UPDRS III: OFF 59, ON 42		duration 12.9 years	DBS-ON only<br>Med-OFF > 12 hours (> 72 hours for DA)	
Liu et al 2006	Static & Dynamic	11 PD	18% female<br>Mean age 53.7 years<br>Mean disease duration 13.1 years<br>Mean stimulation duration 26 months<br>Mean UPDRS III: OFF 42, ON 26	n/a	n/a	*Quiet standing sway:*<br>5 trials 30s<br>*Gait initiation:*<br>self-paced voluntary stepping, 5 trials in each of 4 conditions (med & stim on/off)<br><br>DBS-OFF > 30 mins<br>Med-OFF > 12 hours	CoP displacement (max. In AP & ML)<br>CoP displacement range (AP, ML)<br>APA amplitude (max. vertical)<br>Amplitudes of AP reactive shear forces (swing & standing foot)<br>APA onset<br>Onset & delay of swing foot lifting
McIntyre et al 2011	Static	5 PD	Sex not reported<br>Mean age 57.6 years	n/a	n/a	*Quiet standing sway:*<br>3 trials 60s quiet standing with DT. DT blocks with n-back + postural stability tasks performed simultaneously<br><br>DBS-/Med-OFF times not reported	Sway Area
Muniz et al 2012	Dynamic	6 PD	33% female<br>Mean age 50.4 years<br>Mean disease duration 13.7 years<br>Mean UPDRS III: OFF 42, ON 24	24 HC	54% female<br>Mean age 51.5 years	*Gait initiation:*<br>self-paced voluntary stepping, 4 trials in each of 4 conditions (Med- & DBS- ON/OFF)<br><br>DBS-OFF > 30 mins<br>Med-OFF > 12 hours	Standard distance (principal component analysis)<br>APA onset<br>APA amplitude (max. in vertical, & AP)
Muniz et al 2010	Dynamic	6 PD	33% female<br>Mean age 50.4 years<br>Mean disease duration 13.7 years	31 HC	65% female<br>Mean age 50.1 years	*Gait initiation:*<br>self-paced voluntary stepping, 4 trials in each of 4 conditions	Standard distance (principal component analysis)<br>APA amplitude (max. in vertical & AP)

			Mean UPDRS III: OFF 35, ON 22			(med & stim on/off) at 2 different post-op time points<br><br>DBS-OFF > 30 mins<br>Med-OFF > 12 hours	
Nantel et al 2012	Static	28 PD	25% female<br>Mean age 60.1 years<br>Mean disease duration 9.6 years<br>20 tremor-dominant, 7 akinetic-rigid, 1 mixed<br>Mean LEDD 876 mg	23 HC; 101 no DBS PD	*HC*:<br>15 female<br>Mean age 56.8 years<br>*Total PD:*<br>29 female<br>Mean age 60.6 years<br>Disease duration 8.2 years<br>UPDRS 33.8	*Quiet standing sway:*<br>3 trials<br><br>DBS-ON only<br>Med-OFF > 12 hours (> 24 hours for DA)	RMS of CoP displacement (AP, ML)<br>Mean velocities (AP, ML)
Oz et al 2023	Static	20 PD	20% female<br>Age range 43-81 years<br>Mean LEDD 550 mg	n/a	n/a	*Quiet standing sway:*<br>3 trials >30s quiet standing; investigates DBS at different frequencies (60-230 Hz)<br><br>DBS-/Med-OFF times not reported	Sway Area<br>Sway PL<br>Standard Deviations (AP, ML)
Patel et al 2021a	Static	10 PD	10% female<br>Median age 65 years<br>Median disease duration 18 years<br>Median stimulation duration 37 months<br>Median LEDD 416 mg<br>Median Med-OFF UPDRS III: DBS-OFF 41, DBS ON 22	17 HC	8 female<br>Mean age 71.2 years	*Quiet standing sway:*<br>35s, EO + EC<br><br>DBS-OFF > 30 mins<br>Med-OFF > 10 hours	Body movement coordination (AP; head/shoulder/hip/knee)

Patel et al 2021b	Static & Dynamic	10 PD	10% female<br>Mean age 66.0 years<br>Mean disease duration 18 years<br>Median stimulation duration 37 months<br>Median LEDD 416 mg<br>Median Med-OFF UPDRS III: DBS-OFF 41, DBS ON 22	42 HC: 25 "young" &<br>17 "older"	*"young":*<br>52% female<br>Mean age 25.1 years<br>*"older":*<br>47% female<br>Mean age 71.2 years	*Quiet standing sway:*<br>35s, EO + EC<br>*Balance perturbations:*<br>200-sec calf vibration sequences, eyes open/closed, DBS ON/OFF with Med OFF<br>DBS-OFF > 30 mins<br>Med-OFF > 10 hours	Spectral power of sway frequencies (AP, ML)<br>Body movement coordination (head-shoulder, shoulder-hip, head-hip, head-knee, shoulder-knee, head-knee)
Patel et al 2020	Static & Dynamic	10 PD	10% female<br>Mean age 65 years<br>Mean disease duration 18 years<br>Median stimulation duration 37 months<br>Median LEDD 416 mg<br>Median Med-OFF UPDRS III: DBS-OFF 41, DBS ON 22	17 HC	47% female<br>Mean age 71.2 years	*Quiet standing sway:*<br>35s, EO + EC<br>*Balance perturbations:*<br>200-sec calf vibration sequences. Compares eyes open/closed, DBS ON/OFF and repetition<br>DBS-OFF > 30 mins<br>Med-OFF > 10 hours	Spectral power of sway frequencies (AP, ML)<br>Torque variance
Sato et al 2022	Static	60 PD	47% female<br>Mean age 60.7 years<br>Mean H&Y 2.4<br>Mean disease duration 12.2 years<br>Mean LEDD 1,464 mg<br>Mean UPDRS III ON 18	n/a	n/a	*Quiet standing sway:*<br>30s, EO + EC<br>DBS-ON only<br>Med-ON only, >60 mins	Sway PL<br>Sway Area<br>Mean velocity
Shivitz et al 2006	Static	23 PD	21% female<br>Mean age 58.4 years<br>Mean disease duration 8.9 years	194 HC	n/a	*Sensory Organisation Test (SOT):*<br>mean of 3x 20-sec trials<br>*Target acquisition with leaning*	Equilibrium Score (ES)<br>Leaning (postural) Movement Velocity<br>Reaction Time

			Mean UPDRS III: OFF 40, ON 21			DBS-OFF 17-23 hours<br>Med-OFF > 12 hours (> 24 hours for DA)	
Szlufik et al 2018	Static	20 PD	40% female<br>Mean age 51.1 years<br>Mean disease duration 11.3 years<br>Mean LEDD 1,380 mg<br>Mean UPDRS III: OFF 34, ON 12	20 PD no DBS	55% female<br>Mean age 56.7 years<br>Mean disease duration 10.4 years	*Quiet standing sway:*<br>EO + EC<br><br>DBS-OFF > 30 mins<br>Med-OFF > 12 hours (> 24 hours for DA)	Sway PL<br>Sway Area<br>Mean velocity (AP, ML)
Temiz et al 2022	Dynamic	19 PD	25% female<br>Mean age 58.8 years<br>Mean disease duration 11.7 years<br>Mean UPDRS III: OFF 38, ON 10<br>Mean LEDD 1,082 mg	33 PD-C	30% female<br>Mean age 57.8 years<br>Mean disease duration 11.0 years	*Gait initiation:*<br>self-paced voluntary stepping, 15-30 trials of 5-6m, with 4 conditions as Med-ON & -OFF both pre- and post-op<br><br>DBS-ON only<br>Med-OFF > 12 hours	APA duration<br>Double-stance phases<br>Maximum CoP displacements (AP, ML)<br>First step length, width & velocity
Vallabhajosula et al 2015	Static & Dynamic	19 PD	16% female<br>Mean age 61.8 years<br>Mean disease duration 13.6 years<br>H&Y > II	n/a	n/a	*Quiet standing sway:*<br><br>2 trials 60s, EO + EC<br><br>*Gait initiation:*<br>5 voluntary trials at self-selected pace, initiation split into 3 phases based on COP<br><br>DBS-OFF > 12 hours<br>Med-OFF > 12 hours	Sway Area<br>CoP displacement range (AP, ML)<br>Mean velocity (AP, ML)<br>RMS of CoP displacement (AP, ML)<br><br>Step time, length & velocity<br>Coefficient of variation (step length, step time and step velocity)

Table S2. Studies investigating the effects of GPi-DBS on posturography (includes studies comparing STN- and GPi-DBS)

**Author & Year** *Site*	**Static/Dynamic**	**Participants**	**Participant details**	**Controls**	**Control details**	**Posturography conditions**	**Posturography metrics**
Brandmeir et al 2018 *GPi vs STN*	Static	26 PD<br>*23 STN*<br>*3 GPi*	23% female<br>Mean age 68 years<br>Mean disease duration 6 years<br>25 bilateral, 3 unilateral DBS<br>Mean UPDRS III 42<br>Mean H&Y 3	n/a	n/a	*Quiet standing sway:*<br>EC<br><br>DBS-OFF not reported<br>Med timing not considered	Sway Index
Brecl Jakob et al 2015 *GPi alone*	Dynamic	13 focal/segmental dystonia	31% female<br>Mean age 60.5 years<br>Mean disease duration 15 years	n/a	n/a	*Push & release test*<br>*Pull test*<br><br>DBS-OFF > 60 mins<br>Med timing not considered	Number of steps during Push/Pull tests<br>First step latency<br>First step duration<br>First step length<br>First step maximal velocity
Brecl Jakob et al 2017 *GPi alone*	Dynamic	13 focal/segmental dystonia	31% female<br>Mean age 60.5 years<br>Mean disease duration 15 years	12 HC	"height-, weight-, and sex-matched"	*Gait initiation:*<br>20 trials 1-2 self-paced steps forwards<br><br>DBS-OFF > 60 mins<br>Med timing not considered	APA duration<br>APA velocity (max.)
Johnson et al 2015 *GPi alone*	Static & Dynamic	10 PD	30% female<br>Mean age 58.8 years<br>Mean disease duration 13.4 years<br>Median stimulation 29.4 months<br>Mean UPDRS III: OFF 34, ON 10<br>Mean H&Y 2.5	9 HC	3 female<br>Mean age 60.7 years	*Quiet standing sway:*<br>with visual fixation; 2x 60s trials each EO & EC<br><br>*Target acquisition w/ leaning:*<br>move CoP to eccentric positions; hold for 1 sec<br><br>DBS-OFF > 30 mins<br>Med-OFF > 12 hours	Sway PL<br>Sway Area<br>Mean velocity<br><br>Target acquisition with leaning

Rocchi et al 2002<br>*GPi vs STN*	Static	9 PD<br>*5 STN*<br>*4 GPi*	Mean age 60.1 years<br>Mean disease duration 14.8 years	10 HC	n/a	*Quiet standing sway:*<br>1-3x trials 60s, EO<br><br>DBS-OFF > 20 mins<br>Med-OFF > 12 hours	Sway Area<br>Mean velocity<br>RMS of CoP displacement<br>Direction of maximal sway
Rocchi et al 2012<br>*GPi vs STN*	Dynamic	29 PD<br>*15 STN, 14 GPi*	17% female<br>Mean age 61.3 years<br>*GPI:*<br>Mean disease duration 12.9 years<br>Mean LEDD 1,306 mg<br>Mean UPDRS III: OFF 51, ON 29<br>Mean H&Y: OFF 3.5, ON 2.9<br>*STN:*<br>Mean disease duration 11.9 years<br>Mean LEDD 1,313 mg<br>Mean UPDRS III: OFF 49, ON 21<br>Mean H&Y: OFF 3.2, ON 2.3	28 HC<br>9 PD control	*HC*:<br>Mean age 62.4 years<br>*PD control:*<br>Mean age 60.3 years<br>Mean disease duration 11.6 years	*Gait initiation:*<br>self-paced voluntary stepping, 3 trials of 2 steps starting with right foot in each of 4 conditions (med & stim on/off)<br><br>DBS-OFF > 30 mins<br>Med-OFF > 12 hours	APA velocity (max.)<br>APA duration<br>CoP displacement (max. & mean)<br>Step length & velocity
St George et al<br>2012<br>*GPi vs STN*	Dynamic	24 PD<br>*13 STN*<br>*11 GPi*	*STN:*<br>23% female<br>Mean age 61.6 years<br>Mean disease duration 12.8 years<br>Mean LEDD 1,349 mg<br>Mean H&Y: OFF 3.3, 2.4 ON<br>*GPi:*<br>9% female<br>Mean age 62.8 years<br>Mean disease duration 14.6 years	17 age-matched HC<br>9 PD control	*HC:*<br>18% female,<br>Mean age 65.6 years<br>*PD control:*<br>22% female,<br>Mean age 60.3 years<br>Mean disease duration 11.6 years	*Balance perturbations:*<br>backward perturbation by forward surface translation<br><br>DBS-OFF > 60 mins<br>Med-OFF > 12 hours<br>Med-ON > 45 mins	APR stability<br>Duration of Tibialis burst<br>Area of Tibialis activity<br>Co-activation index

			Mean LEDD 1,412 mg<br>Mean H&Y: OFF 3.8, ON 3.1		Mean LEDD 1,412 mg<br>Mean H&Y: OFF 3.0, ON 2.1		
St George et al<br>2015<br>*GPi vs STN*	Dynamic	29 PD<br>*13 STN*<br>*11 GPi*	*STN:*<br>18% female<br>Mean age 62.0 years<br>Mean disease duration 13.3 years<br>Mean LEDD 1,349 mg<br>Mean UPDRS III: OFF 46, ON 23<br>*GPi:*<br>10% female<br>Mean age 62.8 years<br>Mean disease duration 15.4 years<br>Mean LEDD 1,412 mg<br>Mean UPDRS III: OFF 51, ON 31	17 HC<br>8 PD control	*HC:*<br>18% female<br>Mean age 65.7 years<br>*PD control:*<br>13% female<br>Mean age 60.0 years<br>Mean disease duration 12.1 years<br>Mean LEDD 1,253 mg<br>Mean UPDRS III: OFF 47, ON 23	*Balance perturbations:* forward perturbation by backward surface translation, 3 trials each at 5cm/26cm/comfortable foot width<br><br>DBS-OFF > 45 mins<br>Med-OFF > 12 hours<br>Med-ON > 45 mins	Number of APAs before foot off<br>Foot off latency<br>CoM displacement at foot-off<br>Number of steps to regain balance<br>First step velocity<br>First step length

Table S3. Studies investigating the effects of VIM-DBS and other thalamic sites on posturography

**Author & Year** *Site*	**Static/Dynamic**	**Participants**	**Participant details**	**Controls**	**Control details**	**Posturography conditions**	**Posturography metrics**
Choi et al 2022 *VIM*	Static	17 ET	29% female,<br>Mean age 69.5 years<br>10 unilateral, 7 bilateral DBS	17 HC	Mean age 68.6 years	*Quiet standing sway:*<br>3 trials 30s<br><br>DBS-/Med-OFF times not reported	Sway PL
Espay et al 2008 *VIM*	Static	2 OT	100% female<br>Mean age 70 years	n/a	n/a	*Quiet standing sway:*<br>2 trials 30s quiet standing, EO + EC on firm and foam surfaces<br><br>DBS-/Med-OFF times not reported	Sway PL<br>Sway Area<br>Spectral power of tremor
Kronenbuerger et al 2009 *VL thalamus*	Static & Dynamic	12 ET	17% female<br>Mean age 64.2 years<br>Mean disease duration 35.5 years<br>8 unilateral, 4 bilateral	25 age-matched HC	n/a	*Sensory Organisation Test (SOT):*<br>3 trials 20s (18 trials total)<br><br>*Tandem gait analysis*<br><br>DBS-/Med-OFF times not reported	Sway Area *(SOT)*<br><br>Stride length<br>Cadence<br>Stance Phase<br>Double Limb Support<br>Mis-steps/min
Ondo et al 2006 *Bilateral VIM*	Static & Dynamic	8 PD, 13 ET	29% female<br>Mean age 72.8 years	n/a	n/a	*Sensory Organisation Test (SOT) on EquiTest:*<br>mean of 3x 20-sec trials<br><br>*Balance perturbations*: AP<br><br>DBS-OFF > 30 mins<br>Med-OFF not reported	EquiTest subscores<br><br>APA onset<br>APA amplitude (mean)
Rocha et al 2021 *FF*	Static & Dynamic	13 PD levodopa-unresponsive gait disturbance	53.7% female<br>Mean age 64.9 years<br>Mean disease duration 12.2 years<br>Mean LEDD 1,156 mg<br>Mean UPDRS III ON 49	n/a	n/a	*Quiet standing sway:*<br>using iSway<br><br>*Gait initiation:*<br>iWalk using MobilityLab	Sway Area<br>Mean velocity<br><br>APA duration, latency & peak<br>First step duration

			Mean H&Y 3.5<br>Mean S&E 42			DBS-/Med-OFF times not reported	APA velocity (max. AP, ML)<br>Gait step duration<br>Gait cycle duration<br>Gait stride length<br>Gait foot elevation<br>Turn velocity<br>Cadence

Table S4. Studies investigating the effects of PPN-DBS on posturography (includes studies comparing STN- or CuN-DBS with PPN-DBS)

**Author & Year**<br>*Site*	**Static/Dynamic**	**Participants**	**Participant details**	**Controls**	**Control details**	**Posturography conditions**	**Posturography metrics**
Bourilhon et al<br>2022a<br>*PPN vs CuN*	Dynamic	6 PD with levodopa-unresponsive FOG and/or falls	17% female<br>Mean age 64.8 years<br>Mean disease duration 11.5 years<br>Mean LEDD 1,456 mg<br>Mean UPDRS III: OFF 44, ON 24	n/a	n/a	*Gait initiation:*<br>15-20 trials 5-6m voluntary self-paced walking<br><br>DBS-OFF > 60 mins<br>Med-OFF not reported	APA duration<br>Turn duration<br>Step length<br>Step width<br>Double stance duration<br>Cadence<br>Walking velocity
Bourilhon et al<br>2022b<br>*PPN vs CuN*	Dynamic	6 PD with levodopa-unresponsive FOG and/or falls	17% female<br>Mean age 64.8 years<br>Mean disease duration 11.5 years<br>Mean LEDD 1,456 mg<br>Mean UPDRS III: OFF 44, ON 24	Sham	n/a	*Gait initiation*<br><br>DBS-OFF not performed<br>Med-OFF > 12 hours	Mean CoP displacement (AP, ML)<br>Step length<br>Step velocity<br>Double stance duration
de Oliveira Souza et al 2016	Static	1 PSP	74 year-old female<br>UPDRS III 54	n/a	n/a	*Quiet standing sway:*<br>3 trials 30s, EO + EC on soft surface<br><br>Med-OFF/DBS-ON only; Med-OFF > 12 hours	Sway Area<br>Mean velocity (ML)<br>Standard Deviations (ML vel. & Sway Area)

Mazzone et al 2016	Static	8 PD with severe postural instability	Sex not reported<br>Mean age 63.8 years<br>Mean disease duration 12.0 years<br>Mean H&Y 3.7	n/a	n/a	*Quiet standing sway*<br><br>DBS-/Med-OFF times not reported	Sway PL<br>Sway Area
Welter et al 2015	Dynamic	6 PD	50% female<br>Mean age 63.3 years<br>Mean disease duration 16 years<br>Mean LEDD 984 mg<br>Mean UPDRS III: OFF 46, ON 20	n/a	n/a	*Gait initiation:*<br>first 2 steps measured<br><br>DBS-OFF not reported<br>Med-OFF > 12 hours	First step velocity<br>First step length<br>Double support stance duration<br>Mean CoP displacement (AP, ML)<br>Braking Index
Yousif et al 2016<br>*PPN & STN*	Static	4 PD	0% female<br>Mean age 61.5 years<br>Mean UPDRS III: OFF 55, ON 29	8 HC	Mean age 65 years	*Quiet standing sway:*<br>120s EO + EC<br><br>STN-DBS-ON/Med-ON throughout; PPN-DBS-OFF not reported	Sway PL

Table S5. Static posturography measures

Variable type	Measure	Raw Data	Units	Definition
Dynamic	Sway Path Length (PL)	GRF or sensor	cm	The total distance of the sway path, calculated using CoP (from GRF) or PoP (from sensors)
Positional	Maximum CoP displacement	GRF	cm	The maximum recorded distance (AP, ML) from centre during the trial
Positional	Mean CoP displacement	GRF	cm	The mean distance (AP, ML) from centre during the trial
Positional	Sway/Ellipse Area	GRF	cm^2	The area enclosed by Sway PL per unit of time; often measured as a 95% confidence ellipse, which includes 95% of all sway points [58]
Dynamic	Mean velocity	GRF or sensor	cm/s	The average sway velocity measured during the trial; path length per time unit
Dynamic	Peak velocity	GRF or sensor	cm/s	The maximum velocity reached during a particular trial, including sway and gait initiation. Measured in AP +/- ML
Positional	Root Mean Square (RMS) of CoP displacement	GRF	cm	The variability of sway around the mean CoP trajectory [75]
Positional	CoP displacement range	GRF	cm	The range of maximal distance between 2 points in an orientation (e.g. ML or AP) during a trial
Positional	Direction of maximal sway	GRF	degrees	Angle at which maximal sway occurs during a trial; measured in degrees, where 0 = ML & 90 = AP
Frequency	Spectral power of sway frequencies (AP, ML)	GRF	Normalised	Concentration of sway at certain frequencies (e.g. 0-4 Hz) in each plane (AP, ML) [64,65]
Frequency	Spectral power of tremor	EMG	mV^2	Energy of movements at different frequencies; uses Fourier's transformation & signal filters [64,65]
Frequency	Spectral power frequency of acceleration (AP, ML)	GRF or sensor	Hz	Power frequency of acceleration in a direction, with 95% confidence [60]
Positional	Standard Deviations	CoP	n/a	The square root of the CoP variance during a trial; measured in AP +/- ML

Dynamic	EquiTest subscores on *Neurocom*	GRF (CP)	Normalised (0-100)	CoG sway score in each SOT condition or overall relative to age-matched population (0 = 'fall', 100 = 'full stability' not achievable in practice) [79]
Dynamic	Equilibrium Scores (ES)	GRF (SOT)	%	Max AP CoG sway, as % of theoretical LoS; expressed as median or mean in each SOT condition, where 100% = no sway, 0% = sway beyond LoS [54]
Dynamic	Equilibrium Ratios (R)	GRF (SOT)	Arbitrary	Ratios of ES scores in different conditions (C1-C6), which represent visual, vestibular and somatosensory contributions to balance; expressed to show contribution of each sensory component (e.g. vision) [54]

Table S6. Dynamic posturography measures

Activity/measure type	Measure	Raw data	Units	Definition
LoS on leaning task	Target acquisition	GRF	%	Accuracy of leaning towards a visual target (with feedback) [72,73]
	Time to acquire target	GRF	s	Time taken to reach target by leaning [72,73]
	Trunk swing angle standard deviation	GRF	degrees	Angle of the trunk during leaning [72,73]
	Unsteadiness	GRF	mm	Distance between subject's actual position and center of the target [50]
	Error	GRF	mm	Mean of '*Unsteadiness'* (see above) across the trial [50]
	Entry count	GRF	number	Number of times subject enters the target circle [50]
	Leaning (postural) Movement Velocity	GRF	degrees/s	Speed of movement during leaning (postural) task
	Reaction Time	GRF	ms	Time taken to begin leaning after task instruction
Gait initiation	APA duration (inc. max across trials)	GRF	m/s	Duration of necessary muscle adjustments (postural phase) prior to gait starting (locomotor phase)
	APA onset	GRF	ms	Time at which preparatory movements for gait initiation are deemed to start; must be beyond a threshold
	APA latency	GRF	ms	Time between vocal prompt and muscle activation for preparatory movements for gait
	APA velocity (inc. max)	GRF	mm/s	Maximum velocity recorded during preparatory movements
	APA amplitude (inc. max & mean)	GRF	% body weight or N	Max and mean force used during gait initiation (APA) or following perturbation, in vertical and/or AP planes

	Foot-off (or first step) latency	GRF	ms	Time between vocal prompt or external perturbation and foot-off (or first step)
	Braking Index	GRF	%	Difference between peak velocity in mid-swing phase and at foot contact; reflects stabilisation ability of postural control [82]
	Onset of swing foot lifting	GRF	ms	Time when GRF of swing foot reaches zero [57]
	Delay of swing foot lifting	GRF	ms	Delay of the swing foot lifting after the onset of APA [57]
	Amplitudes of reactive shear forces in AP	GRF	N	Size of forces acting in opposite directions between foot and surface on contacting (swing foot) or leaving (standing foot) the ground [57]
	Standard distance	GRF	PCCs	Algebraic (calculated using PCA) difference between observed participant gait and 'normal' gait, using healthy control data [67,68]
	Standard gait cycle dynamics	n/a	n/a	This includes step/cycle duration, (first) step/stride length, duration, velocity & width, cadence, turn velocity, double-stance support duration, turn velocity and foot elevation. Also includes tandem gait (e.g. number of mis-steps)
	Coefficient of variation	GRF	%	Variability of step length, step time and step velocity in relation to the mean values [59]
Balance perturbations	Body movement coordination (mean)	3D-Motion with body markers	%	Degree of synchronicity between 2 different body segments (1 = perfect synchronicity), reflecting postural strategy (e.g. head-shoulder, shoulder-hip) [39]
	Stability Index (SI)	Biodex system	Arbitrary	Computerised representation of platform displacement, measured as: overall; AP; ML. The closer to 0 the SI, the more stable the posture; high SI is less stable [70]
	Falls Risk Index (FRI)	Biodex system	Arbitrary	Computerised measure of falls risk. Results can be absolute or normalised, inc. age-adjusted [70]
	Torque variance	GRF	N	Measurement of energy used, calculated using changes in CoP [65]
	APR stability	GRF	mm^2	The difference between the actual CoP displacement and the projected CoM location [78]
	No. of APAs before foot-off	GRF	Count (or % of trials)	The number of APAs detected before foot-off and a step is made; reflects delayed stepping (normal = 0)
	CoM displacement at foot-off	GRF	mm	Distance of CoM ahead of CoP when foot-off occurs

	Duration of Tibialis burst	EMG	ms	Duration of Tibialis activity during APR following perturbation [78]
	Area of Tibialis activity	EMG	% of MVC	Area of Tibialis activity during first 75 ms of APR [78]
	Co-activation index	EMG	ms	Duration of antagonist (Gastrocnemius) activity above baseline during Tibialis burst [78]

APA - Anticipatory Postural Adjustments; APR - Automatic Postural Response; CoG – Centre of Gravity; CoM – Centre of Mass; CoP – Centre of (foot) Pressure; CP – computed posturography; CuN – cuneiform nucleus; DA – Dopamine Agonists; DBS – Deep Brain Stimulation; DT – Dual Task; EMG – electromyography; ES – Equilibrium Score; ET - Essential Tremor; EC – Eyes Closed; EO – Eyes Open; FF – Field's of Forel; FoG – Freezing of Gait; GM – gastrocnemius; GPi – Globus Pallidus internus; GRF – Ground Reaction Force; HC – Healthy Control; H&Y – Hoehn & Yahr scale; LEDD – Levodopa Equivalent Daily Dosage; LoS – limits of stability; mm – millimetres; ms – milliseconds; mV – millivolt; MVC – Maximal Voluntary Contractions; N – Newtons; OT – Orthostatic Tremor; PCA – Principal Component Analysis; PCC – Principal Component Coefficients; PD – Parkinson's Disease; PL – Path Length; PoP – Position of Pelvis; PPN – PedunculoPontinue Nucleus; PSP – Progressive Supranuclear Palsy; RMS- Root Mean Square; s – second; S&E – Schwab & England; SOT – Sensory Organisation Test; STN – Sub-Thalamic Nucleus; TA – tibialis anterior; VL - venterolateral
